# Preservation Brow Lift (003): 18 Years of Experience With a Limited Incision, Nonendoscopic, Corrugator-Sparing Technique for Correction of Brow Ptosis

**DOI:** 10.1093/asjof/ojaf175

**Published:** 2026-01-02

**Authors:** Nabil Fanous, Kristina Zakhary, Rawan Arif, Dana Al-Majid

## Abstract

**Background:**

Endoscopic brow lift is presently the procedure of choice for forehead lifting. It includes brow-depressor muscle resection, utilizes 4 to 5 incisions, and uses an endoscope. A recent trend among patients interested in any aesthetic surgery is to seek less invasive interventions while maintaining acceptable results.

**Objectives:**

A preliminary report of a short (45 min), minimally invasive, and safe brow lifting surgical technique with durable results, titled preservation brow lift “003,” is presented as a possible mini-invasive alternative to the conventional endoscopic brow lift.

**Methods:**

The preservation brow lift “003” name refers to “0” muscle resection, “0” endoscope use, and “3” short incisions. All patients who underwent preservation brow lift “003,” by a single surgeon (N.F.) over an 18-year period, were reviewed retrospectively. In this article, the forehead surgical anatomy is first presented in a simplified and practical manner. Secondly, the step-by-step surgical approach is revealed. Thirdly, the results are analyzed, and the examples are demonstrated.

**Results:**

There were a total of 381 cases reviewed. The mean age was 56.5 years. The mean follow-up period was 20.8 months. The mean operative time was ∼45 min. The overall surgical outcome was favorable. There were no major complications. There were no incidences of temporal branch paresis or paralysis. There were no cases of hematomas necessitating a return to the operating room. Five hypertrophic scars (1.3%), 6 cases of dehiscence of incisions (1.5%), 2 cases of persistent scalp paresthesia (0.5%), and 9 cases of temporary scalp paresthesia (2.4%) were encountered. Finally, 2 cases of partial relapse of brow ptosis (0.5%) were revised. The majority of patients (93%) reported being satisfied (62%) or very satisfied (31%) with their overall results, according to their feedback at the most recent visit.

**Conclusions:**

The preservation brow lift “003” is a short, muscle-preserving, and safe technique for forehead lifting, consistently producing long-term results, with limited adverse events. It deserves to be considered as an alternative to the endoscopic brow lift approach.

**Level of Evidence: 4 (Therapeutic):**

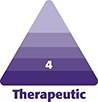

Numerous techniques had been developed to address upper facial rejuvenation before the widespread adoption of the endoscopic brow lift.^1-10^ The latter rapidly grew in popularity and became the preferred procedure since the turn of the century, producing sustainable results. Additionally, the use of neurotoxins in combination with forehead surgical techniques has become more common.^8^

Nowadays, the current aesthetic culture favors minimally invasive approaches that still yield worthwhile results. Endoscopic brow lifting, although less invasive than traditional open procedures, has some disadvantages that motivate the search for an alternative, less invasive technique. For example, it requires expensive advanced endoscopic systems, imaging equipment, and specialized surgical instruments. In addition, resection or excision of the corrugator muscle, as adopted by many endoscopic approaches, may increase the potential for certain risks.

In this paper, we introduce a novel approach, known as the preservation brow lift “003.” The first “0” refers to no muscle resection being performed, the second “0” refers to no endoscope use, and the number “3” refers to the number of short 2 cm incisions required for access. This technique is a muscle-sparing, long-lasting, safe, and nonresource-demanding approach that consistently delivers satisfactory results.

A short preliminary refresher review of the anatomy of the forehead is included with the goal of simplifying the practical understanding of the intricate maneuvers of the preservation brow lift “003” technique.^11-14^

The purpose of this publication is to introduce an alternative to the traditional endoscopic forehead lifting technique: the preservation brow lift “003.” It is a short, corrugator-sparing, low-risk, and resource-sparing technique, using a small number of short incisions and delivering long-lasting, reliable results.

## SIMPLIFIED FOREHEAD ANATOMY

**Figure 1.**
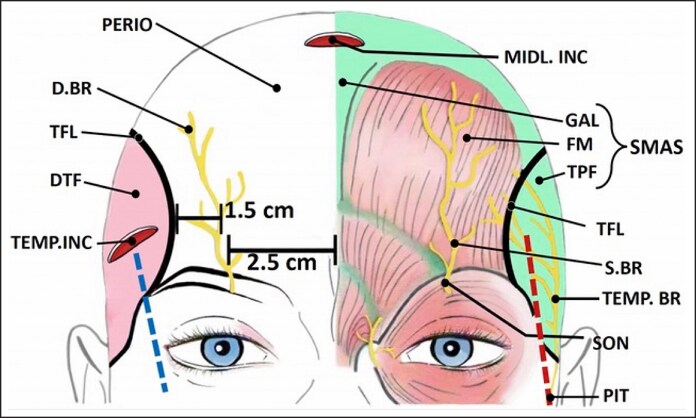
Simplified forehead anatomy, showing the locations of the different structures and landmarks. Left half of forehead (from up to down): midline incision (MIDL.INC); superficial-musculo-aponeurotic system (SMAS); galea aponeurotica (GAL); frontalis muscle (FM); temporo-parietal fascia (TPF); temporal fusion line (TFL); superficial branch of the supraorbital N. (S.BR); temporal branch of the Facial N (TEMP. BR); supraorbital nerve (SON); Pitanguy line (PIT). Right half of forehead (from up to down): periosteum (PERIO); deep branch of the supraorbital N. (D.BR); temporal fusion line (TFL); deep temporal fascia (DTF); temporal incision (TEMP.INC).

The forehead has 1 central and 2 lateral temporal compartments, separated bilaterally by the temporal fusion lines at the border of the temporal fossa (Figure 1; TFL).

### Soft Tissue Envelope

The forehead soft tissue envelope is essentially made up of 3 layers (Figure 1):

1. Skin and subcutaneous tissue.
2. Superficial musculo-aponeurotic system (Figure 1; SMAS): Its central part is comprised of the “galea aponeurotica” investing the frontalis muscle (Figure 1; GAL, FM); its lateral temporal part, the “temporo-parietal fascia” (Figure 1; TPF), is comprised of an investing fascia and mimetic muscles.
3. Periosteum (Figure 1; PERIO): It covers the central forehead. As it reaches each temporal fusion line laterally (Figure 1; TFL), it splits into 2 fibrous layers embracing the temporalis muscle:
  - Superficial fibrous layer that continues laterally over the muscle as the deep temporal fascia (Figure 1; DTF);
  - A deeper fibrous layer forming the periosteum lining the floor of the temporal fossa.

For the purpose of simplifying the coming description of the preservation brow lift “003” technique, the central periosteum and the lateral deep temporal fascia may be considered as 1 continuous layer.

### The 3 Dangerous Areas of the Forehead

The surgeon should be conscious of the following 3 forehead areas in order to avoid serious nerve damage:

1. The exit of the supraorbital nerve (Figure 1; SON): from a foramen at the level of the superior orbital rim, ∼2.5 cm from the midline (in ∼10% of cases, it exits from the frontal bone within 1 cm above the superior orbital rim). This nerve divides into 2 branches: superficial (Figure 1; S.BR) and deep (Figure 1; D.BR).
2. The pathway of the deep branch of the supraorbital nerve (Figure 1; D.BR): some surgeons are unfamiliar with this important nerve, which theoretically might be injured with the paramedian incisions used in some forehead lift approaches. This deep branch travels superiorly, running over the periosteum, ∼1.5 cm parallel and medial to the TFL. It supplies sensation to the parietal scalp.
3. The pathway of the temporal branch of the facial nerve (Figure 1; TEMP.BR): along the Pitanguy line, drawn from the attachment of the lobule to 1.5 cm above the lateral eyebrow (Figure 1; PIT, red dashed line). This nerve runs across the middle third of the zygomatic arch, then through the temporo-parietal fascia in the temples.

## METHODS

This study retrospectively reviewed all patients who underwent the preservation brow lift “003” technique by a single surgeon (N.F.) over an 18-year period (July 2006 to July 2024). Sociodemographic and technical details were noted. Intraoperative and postoperative complications were recorded. Postoperative visits were arranged at least at 1 day, 1 week, and 1 month, then every 5 months thereafter. Patients who were not followed for at least 5 months were excluded from this study. Patient satisfaction was recorded on a 3-point scale as either very satisfied, satisfied, or not satisfied at each postoperative clinic visit.

### The Preservation Brow Lift “003” Surgical Technique

#### Preoperative Marking

The following 4 important surface landmarks are first marked bilaterally as follows:

1. The supraorbital nerve exit point (Figure 1; SON): marked by palpating the supraorbital notch bilaterally. Then, as an extra precaution, an additional point is marked at 2.5 cm from the midline at the level of the superior orbital rim. Those 2 marked dots are to be avoided during forehead dissection.
2. The temporal branch of the facial nerve pathway (Figure 1; TEMP BR): following the “Pitanguy line” (Figure 1; PIT)
3. The 2 temporal fusion lines (Figure 1; TFL): representing the fusion of the periosteum and SMAS aponeurosis to each other and to the bone, along the border of the temporal fossa.
4. The 3 mini-incisions: (1) midline horizontal 2 cm incision (Figure 1; MIDL.INC): positioned at ∼2 cm behind the hairline; (2, 3) bilateral temporal oblique 2 cm incisions (Figure 1; TEMP.INC): positioned at ∼2 cm posterior to the temporal hairline. Each of these 2 oblique incisions is made perpendicular to an imaginary line (Figure 1; blue dashed line) starting at a point 1 cm lateral to the lateral canthus and running superiorly and laterally, at about a 45° angle, to arrive perpendicular to each temporal incision.

#### Preoperative Botulinum Toxin

Two to 4 weeks before the procedure, botulinum toxin Type A (30 units) is injected into the brow-depressor muscles (namely the corrugator, the depressor supercilii, the procerus, and the lateral orbicularis) to help prevent the contraction of such muscles during the early postoperative period, when the adherence of the mobilized forehead flap to the periosteum is vulnerable.

#### Anesthesia

Intravenous sedation was used with the bispectral index monitor (BIS; Medtronic, Dublin, Ireland; using forehead sensors). The 3 forehead compartments, as well as the incision sites, are infiltrated with ∼15 cc of a 1% solution of lidocaine with 1:200,000 epinephrine, including posterior scalp infiltration of a circular perimeter, ∼2 inches (5 cm) posterior to the 3 incisions.

#### The Central Forehead Surgery

The horizontal 2 cm midline incision (Figure 1; MIDL.INC) is made down to the bone, through the skin, subcutaneous tissue, SMAS (galea aponeurotica), and periosteum, using a number 15 blade. Electrocautery is used for hemostasis.

**Figure 2.**
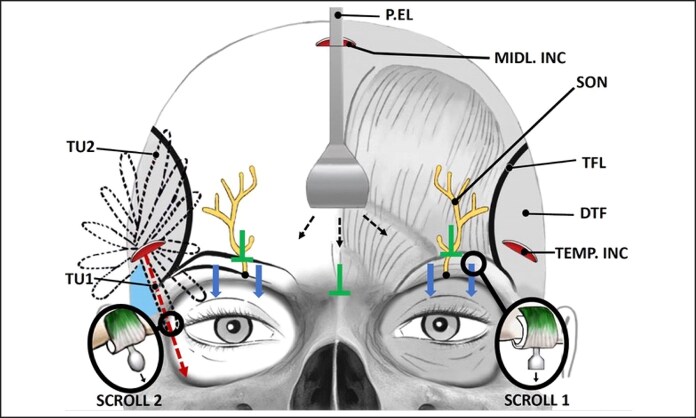
Surgical technique of preservation brow lift “003.” Left half of forehead (from up to down): the periosteal elevator (P.EL) is inserted through the midline incision (MIDL.INC) to dissect the central forehead in the subperiosteal plane; as the periosteal elevator reaches the superior orbital rim, it perforates the 2 sheets of periosteum and SMAS aponeurosis (SCROLL 1), on both sides of the supraorbital nerve, as they rotate around the superior orbital rim; location of the supraorbital nerve (SON); location of the temporal fusion line (TFL); location of the deep temporal fascia (DTF); the temporal incision (TEMP.INC) is made down to the deep temporal fascia; right half of forehead (from up to down): the first sub-SMAS tunnel (TU1) is performed by advancing the blunt dissector toward a point 1 cm lateral to the lateral canthus (red downward arrow), first sliding over the deep temporal fascia, then crossing over the lateral orbital rim and perforating the SMAS aponeurosis as it rotates around the lateral orbital rim (SCROLL 2); then, multiple additional tunnels (TU2) are made close to each other to release the orbital corner, then to release the temporal fusion line and the area around the upper auricle. These extra tunnels are then connected.

The periosteum is dissected in the subperiosteal plane across the central frontal compartment, between the 2 TFL. This is performed using a flat, sharp forehead periosteal elevator (Figure 2; P. EL), with the nondominant hand positioned 2 cm above the superior orbital rim to control and limit the extent of the dissection at this level.

Then, the periosteal elevator is further advanced inferiorly on both sides of the marked supraorbital notch (Figure 2; blue downward arrows), crossing the superior orbital rim. This step involves advancing the periosteal dissector repeatedly, in a back-and-forth seesaw manner, across the superior orbital rim, lateral and medial to each supraorbital notch, in a straight inferior direction, slowly perforating the scroll of the 2 sheets of periosteum and SMAS aponeurosis (Figure 2; SCROLL 1) as they rotate around the superior orbital rim. During this perforating maneuver, the nondominant hand is positioned just below the orbital rim to prevent the periosteal elevator from accidentally perforating the upper eyelid skin. This advancement is stopped when the tip of the dissector is felt under the skin of the upper lid.

Once this perforating maneuver is done bilaterally on both sides of each supraorbital nerve (Figure 2; the 2 blue arrows), the periosteal elevator is then positioned ∼1.5 cm above the area of the supraorbital notch (Figure 2; green inverted-T) and used to forcefully lift up the periosteum away from the bone (toward the O.R. ceiling). This localized periosteal lift will lead to the release of the periosteum at the supraorbital notch, without damaging its nerves and vessels.

Finally, the subperiosteal dissection is extended inferiorly in the midline into the region of the glabella and root of the nose. The periosteum there is further loosened by lifting it up away from the bone (Figure 2; midline green inverted-T). As for the brow depressors, they are spared and not resected.

By now, all of the central forehead should be loose along both superior orbital rims but held laterally at the 2 TFLs (Figure 2). Releasing both TFL, as will be demonstrated later, will help to achieve the main goal of the preservation brow lift “003”: the superior and superolateral mobilization of the forehead layers as 1 unit.

#### The Temporal Forehead Surgery

The temporal incisions (Figure 2; TEMP.INC) are then performed. Each is deepened through skin and SMAS (temporo-parietal fascia) till a bluish shiny sheet is unveiled: the deep temporal fascia (Figure 2; DTF). If this layer does not look shiny, further SMAS tissue layers need to be penetrated.

Then, using a flattened, beaver-tail liposuction cannula as a blunt dissector, the dissection is carried out in the plane overlying this shiny deep temporal fascia (beneath the temporo-parietal fascia containing the temporal branch of the facial nerve), in the form of multiple tunnels (Figure 2; TU1, TU2), made close to each other, then connected later.

The first tunnel (Figure 2; TU1) is performed by advancing the blunt dissector forwards and downwards toward a point 1 cm lateral to the lateral canthus (Figure 2; red downward arrow). Firstly, the blunt dissector slides over the deep temporal fascia. Then, as it reaches the lateral orbital rim (felt under the blunt dissector as a bump on the road), it crosses over it in a back-and-forth seesaw manner till it perforates the scroll of SMAS aponeurosis that rotates around the superior orbital rim (Figure 2; SCROLL 2) and is felt under the skin of the upper lid by the waiting nondominant hand. Another 1 or 2 tunnels are repeated superior to this first tract to free the orbital rim corner.

As explained before, the periosteum in the central forehead may be considered as a continuous layer with the deep temporal fascia in the lateral forehead. Therefore, we now have 2 dissected pockets: the central one that is under the periosteum, whereas the temporal one is above the continuation of that periosteum. In other words, the central and lateral pockets are in different planes and can only be connected by fully breaking the vertical blocking fibrous walls (the 2 TFL, Figure 2) between them. To achieve this, multiple tunnels (Figure 2; TU2) are made repeatedly in a horizontal direction with the blunt elevator to perforate the TFL. Then, these tunnels are connected by placing the blunt dissector in each one of them and lifting it up (away from the bone) to tear the remaining fibrous tissue between the tunnels. If this is not successful, curved Mayo scissors are introduced through the temporal incision, then made to slide over the smooth deep temporal fascia, then over bone, until they reach the area of the persistent fibrous adhesion. At this point, the scissors are opened and advanced, on the bone, on both sides of each adhesion before cutting it. This is repeated till the whole orbital rim corner and the TFLs are freed.

Additionally, extra perforating tunnels are done beyond the TFL, circling superiorly and posteriorly around the upper auricle. These tunnels are then connected easily by rotating the blunt dissector across them. This further dissection facilitates the posterior mobilization of the forehead and scalp flap.

Finally, there is a crucial fact to highlight. By the end of the above temporal dissection, a triangular area above the zygomatic arch is completely avoided (Figure 2; light blue triangle, lateral to the first track TU1), therefore minimizing the risk of injuring the temporal branch of the facial nerve as it crosses the middle third of the zygomatic arch and continues into the temporo-parietal fascia.

At this point, the goal of this forehead lift technique should be finally achieved: the mobilization, in superior and superolateral directions, of the forehead layers.

#### Forehead Flap Fixation

A 1.1 mm hole, placed 1 cm above the anterior border of the midline incision, is drilled manually in the frontal bone using a manual or electric microdrill (by Synthes or Marina Medical). A screw 1.5 mm in diameter and 4 mm long, is inserted into this tiny hole and is rotated to go deeper in it, but is stopped at ∼1 mm from the surface of the bone to allow space for a suture to go around it. A 2.0 Maxon suture (polyglyconate copolymer; Medtronic, Dublin, Ireland) is passed through 3 layers (dermis, SMAS, and periosteum) at the center of the anterior border of the incision. Then, a second identical suture is done next to this first one to make sure this double stitch is solid enough to lift the central forehead flap. Then, the suture is passed around the screw and tied, and the screw is further tightened.

In the temporal incisions, the same double-stitch suture is used at the center of the anterior border of each incision, passing through 2 layers (dermis and SMAS), then tied to the deep temporal fascia and the muscle underneath, pulling the temporal flap in a superolateral direction.

Minimal cutaneous bleeding around the incisions is addressed with fine electrocautery. Occasional active bleeding in the temporal area is controlled with manual pressure by the assistant over the vascular area around the upper lateral orbital corner for at least 2 continuous minutes. We have never encountered persistent severe bleeding.

All incisions are closed with metal clips, which are removed 2 to 3 weeks later. Steri-strips (3M, Saint Paul, MN) and sticky foam are used to cover all the central forehead and are left on the face for 7 days. A small dependent drain (6 French catheter) is inserted into each temporal incision (and pulled out the next morning). A light compression dressing is applied as a final step.

Supplemental Figure 1 as well as the included Video depict the main consecutive surgical steps in the operating room.

#### Postoperative Maintenance

Although a botulinum toxin Type A injection is administered 2 to 4 weeks preoperatively, patients are advised to receive a second injection of the brow-depressor muscles ∼2 months after surgery—that is, ∼3 months after the initial preoperative injection. This additional injection helps maintain the weakness of the brow-depressor muscles during the early, vulnerable postoperative period and until the forehead flap has securely adhered to the underlying tissues. Following this, botulinum toxin Type A injections are typically administered twice annually.

## RESULTS

This study retrospectively reviewed a total of 381 cases who underwent the preservation brow lift “003” technique. Although this technique was started around 18 years ago by the senior author (N.F.), out of 381 cases, a majority of 279 (73%) were performed over the last 10-year period. The mean age in this series was 56.5 years (range, 32-75 years). The mean follow-up period was 20.8 months (range, 5 months to 18 years; Table 1). The average duration of the operative procedure was ∼45 min (Table 1).

**Table 1.** Patient Demographic Characteristics

	*n* (%) or mean ± SD/median (IQR)
Total	381 (100)
Female	341 (89.5)
Male	40 (10.5)
Smoking	99 (25.9)
Mean age (years)	56.5 ± 10.0
Follow-up duration (months), mean	20.8
Follow-up duration (months)	12 (8-24)
Operative time (min)	45 ± 6

IQR, interquartile range; SD, standard deviation.

The majority of patients (93%) reported being satisfied (62%) or very satisfied (31%) with their overall results, according to their feedback at the most recent visit.

There were no instances of temporal branch paresis or paralysis, nor were there any cases of hemorrhage requiring a return to the operating room. Three patients had small unilateral temporal hematomas (0.79%), noted on postoperative Day 1, all of which were effectively expelled out (through the port of the French drain) by applying pressure on the area. Subsequently, a small drain was reinserted and removed the following day. Five patients had hypertrophic scars (1.3%) in one of the incisions, which were revised under local anesthesia. Six patients had wound dehiscence (1.6%) caused by trapped hair and were addressed by revision under local anesthesia. There were 2 cases of unilateral persistent partial scalp paresthesia (0.52%), and 9 cases of temporary scalp paresthesia (2.4%) that lasted beyond 2 months (1 lasting up to 15 months). Finally, there were 2 cases of partial relapse of brow ptosis (0.52%) that required reoperation at 7 and 10 years after their initial surgery (Table 2).

**Table 2.** Postoperative Complications

Complication	*n* (%)
Temporal branch paresis/paralysis	0 (0)
Persistent unilateral scalp paresthesia	2 (0.52)
Temporary scalp paresthesia	9 (2.4)
Hypertrophic scar	5 (1.3)
Wound dehiscence	6 (1.6)
Partial relapse of brow ptosis	2 (0.52)

There were no other reported aesthetic complications, such as medial brow elevation, lateral brow spreading, glabellar depressions (inter-brow soft tissue defects), asymmetric brows, or distorted asymmetrical forehead expressions.

Postoperative maintenance involving periodic botulinum toxin injections, approximately twice a year, was recommended to patients, although we have observed effective results with less frequent injections. At a mean period of follow-up of 2 years for the cohort, 82% of patients were complying with the regular injections.

**Figure 3.**
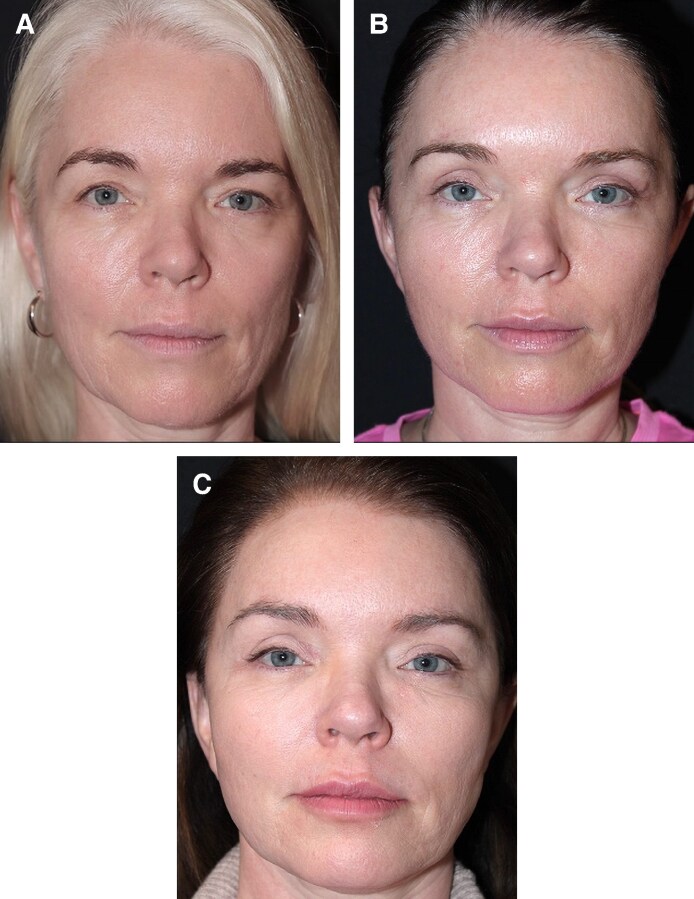
A 53-year-old female patient, (A) preoperative view, (B) 5 months following preservation brow lift combined with facelift and blepharoplasty, and (C) postoperative view at 1 year. Notice the slight improvement of the brows’ position (brow-to-lash distance) over time.

**Figure 4.**
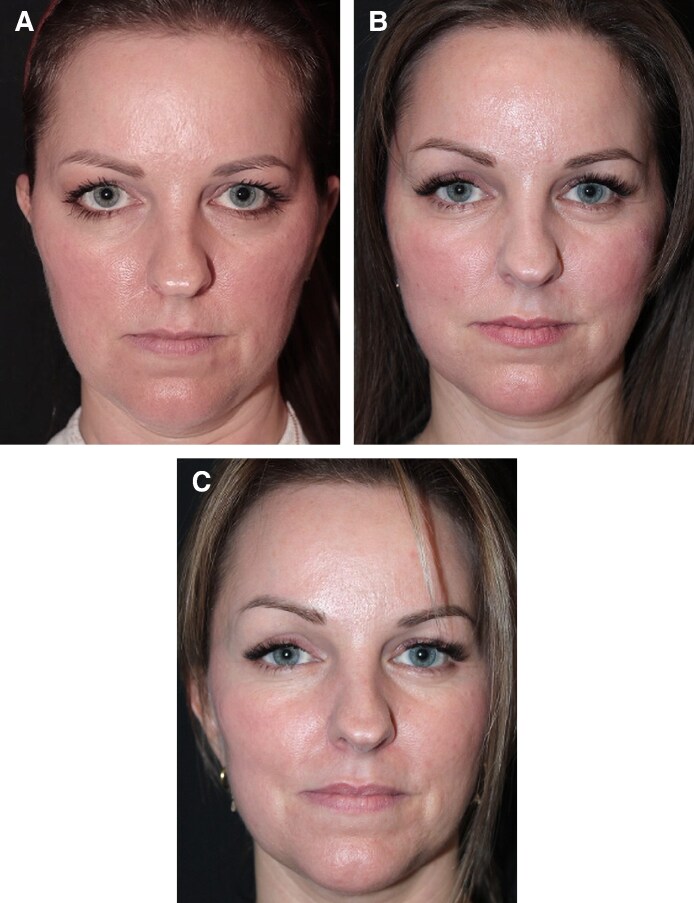
A 44-year-old female patient, (A) preoperative view, (B) postoperative view 10 months following preservation brow lift combined with facelift, and (C) postoperative view at 1 year and 3 months. Notice the improvement of the brows’ position over time.

**Figure 5.**
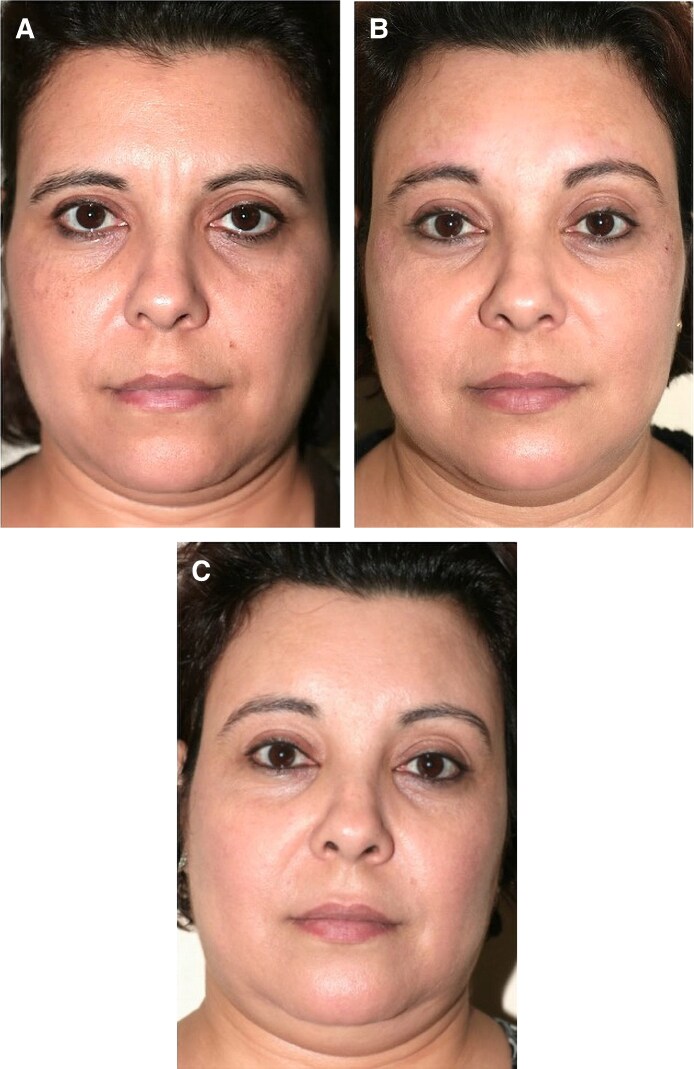
A 42-year-old female patient, (A) preoperative view, (B) postoperative view at 3.5 years following preservation brow lift, and (C) postoperative view at 4.7 years. Notice the improvement of the brows’ position over time.

**Figure 6.**
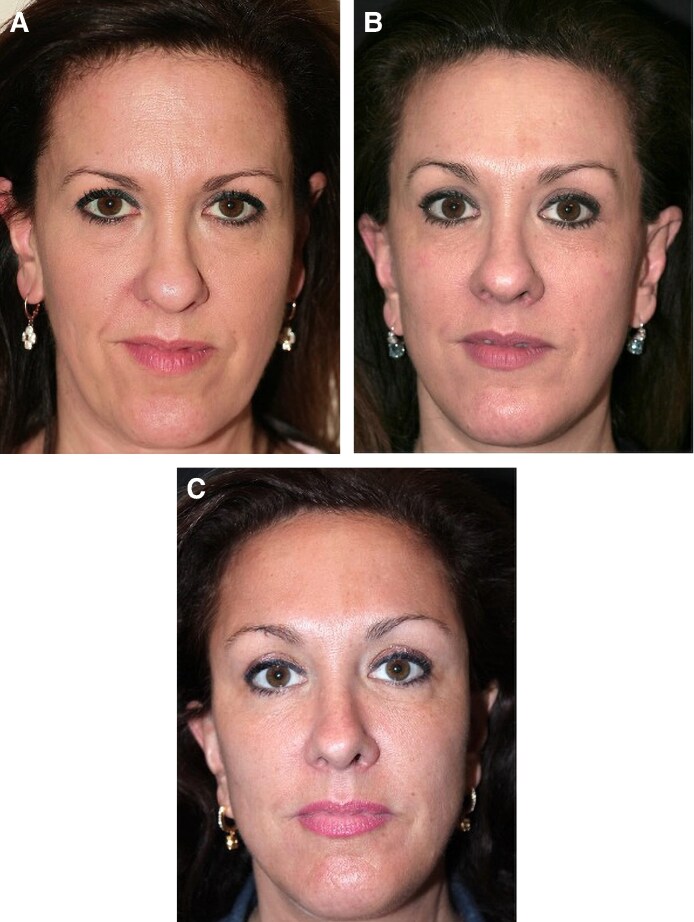
A 55-year-old female patient, (A) preoperative view, (B) postoperative view 11 months following preservation brow lift combined with facelift and blepharoplasty, and (C) postoperative view at 4 1/2 years. The patient had tip narrowing surgery in between. Notice the improvement of the brows’ position over time.

**Figure 7:**
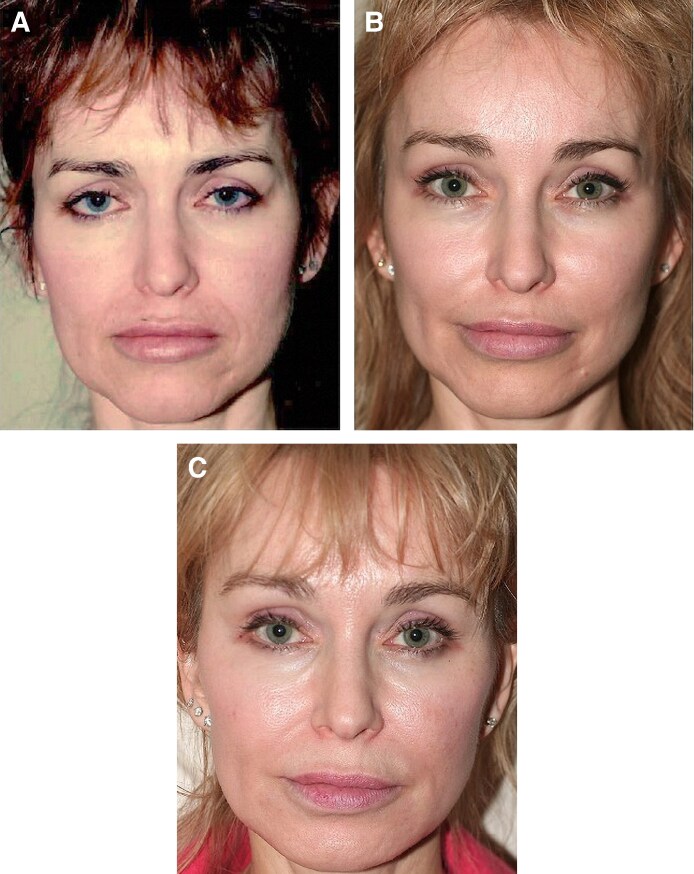
A 40-year-old female patient, (A) preoperative view, (B) 6-year postoperative view following preservation brow lift and lower blepharoplasty, and (C) postoperative view at 15 years. The patient also had a mini facelift 4 years before this photograph, but no further intervention to the forehead. The improvement of the brows’ position has been very well sustained over 15 years.

Figures 3-7 provide preoperative and postoperative examples of the outcomes achieved in this study.

## DISCUSSION

In this paper, we introduce the novel preservation brow lift “003” as a muscle-preserving, durable, safe, and fast technique, without the need for an endoscope or cumbersome instrumentation.

The 3 key principles of the preservation brow lift “003” involve sparing the brow-depressor muscles, making a small number of short incisions (three, 2 cm each), and avoiding endoscope usage.

Several other techniques have been previously developed for brow lifting and were considered the workhorses of forehead lift surgery in the past, such as the coronal approach and its variations (hairline and trichophytic types) and the direct or mid-forehead brow lifts. They were replaced to a great extent by the endoscopic forehead lift because of its better outcome and fewer complications.^15-20^

Compared with the endoscopic brow lift, the preservation brow lift “003” technique offers advantages such as muscle sparing (thus avoiding the potential undesirable late sequelae of muscle resection), the shorter duration of surgery, the use of less short incisions, and the elimination of costly and cumbersome equipment. Additionally, the adoption of a single midline incision has 2 advantages: (1) decreasing the chance of injury of the deep branch of the supraorbital nerve, which is theoretically more at risk with paramedian incisions; and (2) in the case of balding male patients, a unique central incision is less obvious as a telltale sign of surgery compared with 3 incisions (1 central and 2 paramedian).

The safety record is quite favorable as well, and the results are satisfactory and long-lasting (Figures 3-7). Postoperative maintenance involving periodic botulinum toxin injections, approximately twice a year, is recommended to patients. Finally, from a patient's perspective, a perceived advantage of the preservation brow lift “003” technique is the use of only 3 short incisions, instead of the 4 to 5 incisions of the traditional endoscopic forehead lifts. Some limitations of this study include its single-center, single-surgeon design and the lack of an objective outcome measure with statistical analysis to quantify changes in the brow-to-lash distance. Nevertheless, the main value of this publication lies in its practical utility, providing the reader with very detailed, step-by-step guidance on performing this safe and durable brow lift technique.

### The Phenomenon of Durability and Continued Enhancement

The senior author (N.F.) has been surprised over the years to consistently notice that, not only the results were quite durable, but, interestingly, they also tended to slightly improve over time in most patients. This enhancement appears as a very slight ascent of the brows (increased brow-to-lash distance), mostly during the first 3 or 4 postoperative years, then seems to slow down or stabilize afterward (Figures 3-7). A potential hypothesis of this delayed enhancement of the results with this technique could be the absence of muscle resection, which could theoretically cause adhesions to the bone, therefore inhibiting further future ascent of the brows.

## CONCLUSIONS

The preservation brow lift “003” is a novel, resource-sparing, muscle-preserving, and short technique for forehead lifting, with reliable and long-lasting results. The overall complication rate in this patient cohort was low, with no major complications (motor nerve paralysis, significant postoperative bleeding necessitating emergency intervention) and a low overall revision rate. The preservation brow lift “003” could be an appealing alternative to the conventional endoscopic forehead lifting technique.

## Supplemental Material

This article contains supplemental material located online at https://doi.org/10.1093/asjof/ojaf175.

## Supplementary Material

ojaf175_Supplementary_Data
